# After Treatment of Cataract Extractions and Iridectomies

**Published:** 1888-01

**Authors:** R. O. Cotter

**Affiliations:** Macon, Georgia


THE ATLANTA MEDICAL AND SURGICAL JOURNAL.

VOL. IV. JANUARY, 1888. No. 11.

### Original Communications.

### AFTER TREATMENT OF CATARACT EXTRACTIONS AND IRIDECTOMIES.

BY R. O. COTTER, M. D., MACON, GEORGIA.

Up to within the past year or so, it was my experience that cataract patients were not allowed to go home until from four to six weeks, and often more, after being operated upon.

After running the risk of sloughing of the cornea within the first three days, they often had a most annoying and tedious iritis follow the operation. My observation is that this was too frequently caused by useless, and even meddlesome, interference in the after-treatment in our anxiety to keep down inflammation.

Instead of allowing nature to heal the wound made by the surgeon's knife, it was the custom to remove the dressing too often, and thus more or less disturb the corneal wound, and then drop in twice daily a solution of atropia. I am thoroughly satisfied I have frequently seen this cause a very severe iritis. Very likely it was the well-known atropia irritation.

The last four cases of extraction I have done—as were two recent cases of iridectomy for acute glaucoma—were managed in the following manner: No antiseptic, except pure water, was used. In crowded hospitals, I suppose antiseptics are necessary. I will say, however, all the instruments were first dipped in alcohol. I use always a 4 per cent. solution of cocaine, and have yet to see any harm from its use here. I employ Graefe's linear extraction. After the operation I immediately drop a 2 grain to the oz. solution of sulphate of atropia into the eye, anoint the lids thoroughly with vaseline, apply soft compresses of absorbent cotton over the closed lids (both eyes) and then apply a firm bandage. Then I put them in bed and tell them they may sit up some in bed the next day, and then let them alone.

They should not be allowed any solid food for three or four days, because the movements of the jaws necessary in chewing will certainly cause a determination of blood to the eye. I visit them in about six hours. If they complain of an accumulation of hot tears within the eyelids, I turn up the bandage and carefully let out the tears by slightly pulling down the lower lid. Otherwise I do *nothing*.

If all goes well, as they usually will if let alone, in about three or four days I change the dressing and carefully examine the eye and then, perhaps (but not always), drop in a 2 grain solution of atropia.

In the four cases referred to (the four last cases I have operated upon), the patients all went by themselves to their homes, ranging from 30 to 75 miles distant, within fourteen days, one of them in eleven days. All were successes.

In the last case, an old man 74 years of age, within 6 hours after the operation there came on severe pain, puffiness of the lids, and flow of hot tears, showing decided and alarming symptoms of beginning sloughing of the cornea. He was given a hypodermic of ¼ grain morphine, the hot tears were let out, and in 4 hours all the symptoms disappeared.

In the cases of iridectomy for glaucoma, where we wish to make a large one, I adopt precisely the same treatment.

To sum up: We should recollect that nature is the real healer of wounds, and that rest is her salve.

